# Different Regions of the Newcastle Disease Virus Fusion Protein Modulate Pathogenicity

**DOI:** 10.1371/journal.pone.0113344

**Published:** 2014-12-01

**Authors:** Sandra Heiden, Christian Grund, Anja Röder, Harald Granzow, Denis Kühnel, Thomas C. Mettenleiter, Angela Römer-Oberdörfer

**Affiliations:** 1 Institute of Molecular Virology and Cell Biology, Friedrich-Loeffler-Institut, Federal Research Institute for Animal Health, Greifswald-Insel Riems, Germany; 2 Institute of Diagnostic Virology, Friedrich-Loeffler-Institut, Federal Research Institute for Animal Health, Greifswald-Insel Riems, Germany; 3 Institute of Infectiology, Friedrich-Loeffler-Institut, Federal Research Institute for Animal Health, Greifswald-Insel Riems, Germany; Virginia Polytechnic Institute and State University, United States of America

## Abstract

Newcastle disease virus (NDV), also designated as Avian paramyxovirus type 1 (APMV-1), is the causative agent of a notifiable disease of poultry but it exhibits different pathogenicity dependent on the virus strain. The molecular basis for this variability is not fully understood. The efficiency of activation of the fusion protein (F) is determined by presence or absence of a polybasic amino acid sequence at an internal proteolytic cleavage site which is a major determinant of NDV virulence. However, other determinants of pathogenicity must exist since APMV-1 of high (velogenic), intermediate (mesogenic) and low (lentogenic) virulence specify a polybasic F cleavage site. We aimed at elucidation of additional virulence determinants by constructing a recombinant virus that consists of a lentogenic NDV Clone 30 backbone and the F protein gene from a mesogenic pigeon paramyxovirus-1 (PPMV-1) isolate with an intracerebral pathogenicity index (ICPI) of 1.1 specifying the polybasic sequence R-R-K-K-R*F motif at the cleavage site. The resulting virus was characterized by an ICPI of 0.6, indicating a lentogenic pathotype. In contrast, alteration of the cleavage site G-R-Q-G-R*L of the lentogenic Clone 30 to R-R-K-K-R*F resulted in a recombinant virus with an ICPI of 1.36 which was higher than that of parental PPMV-1. Substitution of different regions of the F protein of Clone 30 by those of PPMV-1, while maintaining the polybasic amino acid sequence at the F cleavage site, resulted in recombinant viruses with ICPIs ranging from 0.59 to 1.36 suggesting that virulence is modulated by regions of the F protein other than the polybasic cleavage site.

## Introduction

Newcastle disease (ND) is a highly contagious infection caused by Newcastle disease virus (NDV) which affects more than 250 species of birds. ND causes high economic losses in the poultry industry worldwide, in particular in chickens and turkeys [1]. NDV, or avian paramyxovirus type 1 (APMV-1), is a non-segmented single-stranded RNA virus that belongs to the genus *Avulavirus* within the family *Paramyxoviridae* of the order *Mononegavirales* [2]. Genome sizes vary between 15,186 (class II genotype I–IV, early isolates), 15,192 (class II genotype V–VIII, late isolates) or 15,198 nucleotides (nt) (class I) [3] which encode the six major proteins nucleoprotein (NP), phosphoprotein (P), matrix protein (M), fusion protein (F), hemagglutinin-neuraminidase protein (HN), and RNA-dependent RNA polymerase (L) [4]. Like other paramyxoviruses, NDV encodes additional gene products, named V and W, which arise from the P gene translated from alternative mRNAs produced by RNA editing during P gene transcription [5]. Based on the severity of clinical signs in chickens, three pathotypes, i.e. lentogenic, mesogenic or velogenic NDV can be distinguished by their intracerebral pathogenicity index (ICPI). Lentogenic strains (ICPI<0.7) induce only mild respiratory signs in young chickens, whereas mesogenic strains (ICPI 0.7–1.5) cause moderate mortality. Infection with velogenic strains (ICPI>1.5) results in diarrhea, hemorrhages, intestinal lesions, respiratory and neurological signs with mortality up to 100% [6]. Besides chickens and turkeys, pigeons can also be infected by APMV-1. In the 1980ies, a disastrous epidemic in pigeons spread over Europe and other countries [7] primarily associated with neurological signs similar to NDV in chickens. One of the first isolated pigeon paramyxovirus-1 (PPMV-1) was the BVC 78 strain that has been responsible for the disease in pigeons since 1981. It was suggested that this virus has spread from Mesopotamia westward, was observed in early spring of 1982 in the Nil delta area, and almost at the same time in Sudan and north of the Mediterranean sea in Italy [8]. Investigation of different PPMV-1 isolates of the 1982 outbreaks in Italy showed a higher hemagglutinin-thermostability, slower hemagglutinin-elution patterns and a considerable antigenic diversity. Most important, however, was the demonstration of a lack of pathogenicity of these PPMV-1 isolates for chickens, whereas morbidity was 80% and mortality 55% after experimental infection of pigeons [9]. Nevertheless, PPMV-1 strains may pose a risk for chickens and turkeys, since virulence of PPMV-1 isolates increases following passages in chickens [10], [11] and a number of ND outbreaks in chickens have been attributed to PPMV-1 [6], [12], [13].

The two surface proteins of NDV are F and HN. HN is involved in virus attachment and release, and F mediates fusion of the viral envelope with cellular membranes. However, the HN protein is also required for fusion by interaction with F [4], [14], [15]. Although virulence of different APMV-1 strains appears to be modulated by both surface proteins, F is known as the main determinant of pathogenicity which was confirmed recently by a systematic study of chimeric NDVs with substitutions of genes between a mesogenic and a velogenic strain [16]. The F protein is a class I transmembrane protein that is synthesized as a precursor protein F_0_. The cleavage of the precursor protein F_0_ into the disulfide-linked subunits F_1_ and F_2_ by cellular proteases is necessary for infectivity of progeny viruses [17]. Mesogenic and velogenic viruses exhibit a polybasic amino acid (aa) motif ^112^(K/R)-R-(Q/K)-(R/K)-R^116^ at the carboxy terminus of F_2_ and a phenylalanine at the amino terminus of the F_1_ subunit (position aa 117) which are substrates for ubiquitously existing furin-like proteases detected in a wide range of cells and tissues, resulting in systemic infections [18]. In contrast, the F protein of lentogenic viruses is characterized by a leucine at position 117 and a monobasic aa motif at the carboxy terminus of F2 ^112^(G/E)-(K/R)-Q-(G/E)-R^116^, resulting in a virus that can be processed only by trypsin-like enzymes restricted to the respiratory and intestinal tract which limits virus replication [19]–[21]. Therefore, the F protein cleavage site is a major determinant of NDV virulence [17]. However, other pathogenicity determinants must exist because differences in NDV virulence can not be explained by the F protein cleavage site only. Importantly, the investigation of 27 PPMV-1 isolates from racing pigeons of different regions of Belgium from 1998 and 1999 showed presence of a polybasic cleavage site but only low to intermediate virulence [22]. In line with this observation, alteration of the monobasic F cleavage site in lentogenic NDV strains La Sota or Clone 30 to a polybasic site resulted in an increase of virulence but not to a velogenic level [23].

Here, we describe the generation of a recombinant NDV on the basis of lentogenic strain Clone 30 that expresses the F surface protein of a mesogenic PPMV-1, specifying the polybasic aa motif ^112^R-R-K-K-R*F^117^ at the F protein cleavage site, which was isolated in 1998 in Germany, instead of the Clone 30 F protein. The resulting virus induced syncytia in cell culture as expected from fusogenic activation of the polybasic F cleavage site, but its ICPI was 0.6, characterizing a lentogenic virus and indicating presence of other virulence-determining regions. For their identification, different regions of the Clone 30 F protein were substituted in different recombinant NDV based on Clone 30 by respective regions of the PPMV-1 protein while maintaining the polybasic F cleavage site. The results of characterization demonstrate that other regions within the F protein besides the proteolytic cleavage site influence pathogenicity.

## Materials and Methods

### Viruses and cells

Lentogenic NDV Clone 30 was obtained from MSD Animal Health, Boxmeer, the Netherlands. Recombinant NDV (rNDV) based on Clone 30 has already been described [24]. The mesogenic PPMV-1 isolate R75/98 (ICPI 1.1) was received from the National Reference Laboratory for Newcastle Disease, FLI Insel Riems.

QM9 (CCLV-RIE 999, quail muscle cells) are a clone of QM7 [25] which were derived from QT6 [26], and were grown in an equal volume mixture of Ham's F12 medium and Iscove's modified Dulbecco's medium containing L-Gln and 10% fetal bovine sera. The cells were obtained from the Collection of Cell Lines in Veterinary Medicine (CCLV). Chicken embryo fibroblasts (CEF), prepared from 10-day-old specific-pathogen-free (SPF) chicken embryos, were maintained in minimum essential medium with NaHCO_3_, Na-Pyruvate, non-essential amino acids and 10% FBS. SPF embryonated chicken eggs (ECE) for virus propagation and cell preparation were purchased from Lohmann, Cuxhaven, Germany and incubated at 37°C and 55% humidity. Furthermore, BSR-T7 cells (BHK 21, clone BSR-T7/5) [27], stably expressing the T7 RNA polymerase, were used to recover infectious virus from cDNA. BSR-T7 cells were grown in Glasgow minimal essential medium supplemented with NaHCO_3_, tryptose phosphate, caseine peptone, meat peptone, yeast extract, essential amino acids and 10% FBS.

### Sequence determination of NDV R75/98 F gene

A 3.9 kb PCR fragment was amplified by one-step-RT-PCR (QIAGEN) using RNA of NDV R75/98 and primers PND75-98MFF 5′-cta tct ttt aat taa ata gtt agt tta cct gtc-3′ (containing restriction site *Pac*I nt 4,458, nt position refers to Clone 30) and PND75-98HNLR 5′-acc cgt gct acg tat tgt atg ttg ttt tcc cc-3′ (containing restriction site *SnaB*I nt 8,364, nt position refers to Clone 30). The resulting fragment was ligated into pGemTeasy vector (Promega) resulting in pGemFHNR75 that was used to determine the complete nucleotide sequence (GATC, Konstanz, Germany) and subsequently for further cloning. Thereafter, sequence was confirmed by Next generation sequencing of the full-length genome as submitted to GenBank (Accession number KJ736742).

### Construction of full-length plasmids

**Figure 1:**
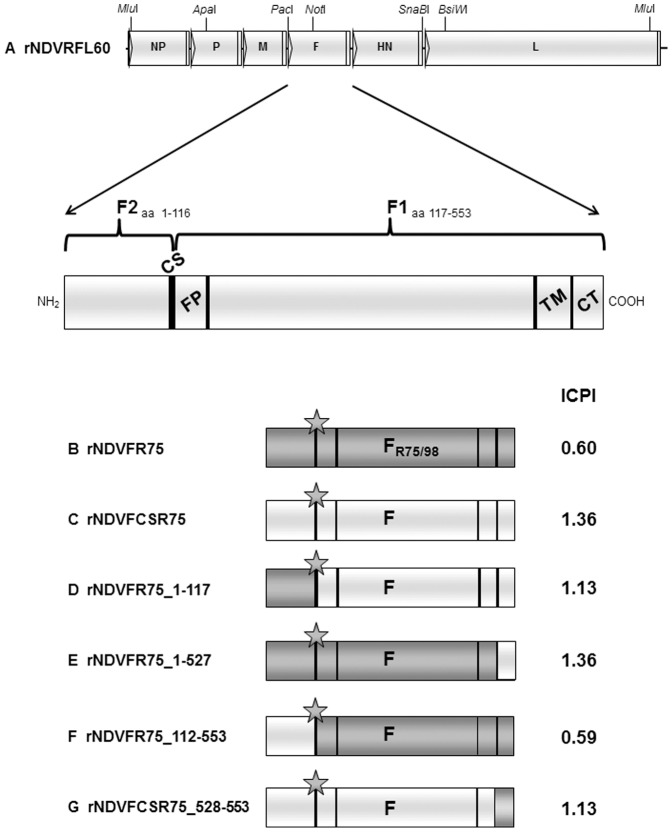
Construction of full-length plasmids. Schematic representation of genome organization of new generated recombinant NDV based on pflRFL60. Sites of restriction enzymes used for different cloning steps are given. Sequence regions raised from R75/98 are in dark grey, sequence regions raised from rNDV are in light grey. ICPI values for each virus are given on the right.

pflNDVRFL60 (Figure 1A) is based on plasmid X8δT [28] containing the full-length genome of NDV Clone 30 (GenBank Acc. No. Y18898) with four artificially introduced restrictions sites (*Mlu*I nt 76, nt 15,039; *Pac*I nt 4,458 and *SnaB*I nt 8,364, nt positions refer to Y18898). Plasmid pGemFHNR75 was cleaved with *Pac*I and *SnaB*I and the resulting insert fragment was used to substitute the corresponding Clone 30 fragment in pfNDVRFL60, resulting in pflNDVFHNR75. To generate a plasmid that carries only the F gene of R75/98 within a Clone 30 backbone, the HN gene of pflNDVFHNR75 was replaced by that of Clone 30 using Phusion-PCR [29] with a megaprimer that was generated by PCR with primers PPHN75CH4F (5′-caa cca aaa agc aat aca cgg gta gaa cgg taa gag agg ccg ccc ctc-3′), PPHN75CH4R (5′-gta cct cac tat cat cca tat ttt ttc tta atg aag aga cta ttg aca ag-3′) and a plasmid containing the Clone 30 sequence resulting in pflNDVFR75 (Figure 1B). To generate a plasmid containing the full-length NDV Clone 30 genome with the F cleavage site of R75/98, an appropriate *Apa*I-*Not*I fragment of the NDV Clone 30 sequence was subcloned and then mutagenized using the QuikChange II XL Site-Directed Mutagenesis Kit (Stratagene) and primer pair MPF3F/R (indicated is the forward primer, the reverse primer (R) is reverse complementary) (5′-cgc caa gag tct gtg act aca tct gga ggg aga aga aag aag cgc ttt ata ggc gcc att att ggc ggt gtg gct c-3′). Finally, the *Apa*I-*Not*I fragment of pflNDVRFL60 was substituted by the mutagenized version, resulting in plasmid pflNDVFCSR75 (Figure 1C). To generate further full-length genome containing plasmids with NDV Clone 30 sequence but chimeric F genes with parts of R75/98 F, different megaprimers were generated by PCR using pflNDVFR75 as template and primers consisting of a 5′end consistent with NDV Clone 30 and a 3′end matching with PPMV-1 R75/98. Primers and resulting megaprimers are given in table 1. These megaprimers were then used to alter the respective sequences within a plasmid containing the *Apa*I-*BsiW*I-fragment of pflNDVRFL60. Substitution of the *Apa*I-*BsiW*I-fragment of pflNDVRFL60 resulted in different full-length genome containing plasmids, named pflNDVFR75_1–117 (Figure 1D), pflNDVFR75_1–527 (Figure 1E), and pflNDVFR75_112–553 (Figure 1F). To construct a full-length genome containing plasmid based on NDV Clone 30 but with the cleavage site and the cytoplasmic tail of R75/98 F protein, a plasmid containing the *Not*I-*BsiW*I-fragment (nt 4953–8852) of NDV Clone 30 (Y18898) was mutagenized using the QuikChange II XL Site-Directed Mutagenesis Kit (Stratagene) and primer pair MPCTF75F/R (indicated is the forward primer, the reverse primer (R) is reverse complementary to given sequence) (5′-gat cag atg aga gcc act aca aga aca tga aca cag atg agg aac gaa gg-3′). Finally, the *Not*I-*BsiW*I fragment of pflNDVRFL60F3 was substituted by the mutagenized version, resulting in rNDVFCSR75_528–553 (Figure 1G).

**Table 1:** Sequences of primers used for Phusion-PCR.

primer	sequence (5′-3′)	length in bp
PP30_75_4488F	tag aaa aaa cac ggg tag aag agt ctg gat ccc ggc tag cac att c	434
PP30_75_4921R	gcc aca ccg cca ata atg gcg cct ata aag cgc ttc ttc ctc ctt act cc	
PP30_75_4488F	tag aaa aaa cac ggg tag aag agt ctg gat ccc ggc tag cac att c	1654
PP30_75_6141R	tgt tgc gcc tttt gct tgt aca tca ggt agc atg cta gaa c	
PP30_75_4857F	ctg tga cta cat ctg gag gga gga gga aga agc gct tta tag gtg cc	1451
PP30_75_6307R	gtc atc tac aac cgg tag ttt ttt ctt aaa tct tcc att gaa cag gtt gtc ag	

### Transfection and recovery of recombinant NDV

To recover recombinant NDV, full-length genome containing plasmid clones were transfected together with helper plasmids pCiteNP, pCiteP and pCiteL expressing the NP, P and L proteins of NDV Clone 30 into BSR-T7 cells using Lipofectamine 2000 (Invitrogen, Karslruhe, Germany) at a DNA∶lipofectamine ratio of 1∶1,5 (µg∶µl) following the manufacturer's instructions. Confirmation of infectious virus recovery and virus propagation was done as already described [24].

### RNA preparation, RT-PCR and sequencing

Viral RNA was isolated from allantoic fluid using Trizol reagent (Invitrogen) according to the manufacturer's instructions. Virus identity was verified by RT-PCR using OneStep RT-PCR Kit (Qiagen) to amplify selected regions. Virus identity was approved by sequencing (Sequencer 3130Genetic Analyzer, Applied Biosystems).

### Western blot analysis

Virions were purified by ultracentrifugation of the respective allantoic fluids through a continuous CsCl gradient (20%–45%) at 27,000 rpm for one hour. After normalizing the total protein levels (10 µg/lane), proteins were separated under denaturing conditions in sodium dodecyl sulphate (SDS)-11% polyacrylamide gels, transferred on a nitrocellulose membrane and incubated with a polyclonal monospecific antiserum against NDV-F (Clone 30) protein [30] followed by incubation with anti-rabbit peroxidase labeled secondary antibody (Dianova, Germany). Proteins were detected by chemiluminescence using SuperSignal West Pico Chemiluminescent Substrate (Pierce) and the Chemi Doc XRS+ imaging system (Bio-Rad). Subsequently, a second Western blot was performed using a monoclonal antibody against the NDV-NP protein, followed by incubation with peroxidase labeled anti-mouse secondary antibody (Dianova, Germany) and detection by chemiluminescence.

### Indirect immunofluorescence assay (IIFA)

Cells were fixed with acetone/methanol (1∶1) 20 h post infection (p.i.) or 72 h post transfection. Detection of recombinant NDV was done using monoclonal antibody HN-10 [13] directed against NDV HN protein and FITC-conjugated goat anti-mouse IgG F(ab)_2_ (Dako, Denmark) or NDV hyperimmune serum (HIS) and FITC-conjugated anti-rabbit IgG (Sifin, Germany).

### Kinetics of viral replication

Kinetics of replication were established in embryonated chicken eggs (ECE), CEF and QM9 cells. ECE were infected with 10^2.3^ TCID_50_ per egg and incubated at 37°C and 55% humidity. ECE with living embryos were euthanized by cooling at the indicated time points. The allantoic fluids were harvested, clarified by centrifugation, and two pools were generated and frozen. After thawing of the frozen samples, virus titers were determined on QM9 cells by indirect immunofluorescence using HIS against NDV and FITC conjugated anti-rabbit IgG (Sifin, Germany). CEF and QM9 cells were infected at a multiplicity of infection (moi) of 0.01 with respective viruses and incubated at 37°C and 3% CO_2_ atmosphere. After 40 minutes, the inoculum was removed and the cell monolayers were washed twice with medium. QM9 cells were additionally treated with citrate buffer saline for 2 minutes to inactivate non-penetrated virus before overlaying with fresh medium. Cells were incubated at 37°C and 3% CO_2_ after adding fresh medium. The supernatants were harvested and frozen at indicated time points in two independent experiments.

### Syncytium-assay

Monolayers of BSR-T7 cells were infected at an moi of 0.01 with respective viruses. After 40 minutes, the inoculum was removed and the cells were washed twice with medium. Then, cells were overlaid with fresh medium and incubated at 37°C and 3% CO_2_ for 24 hours. Cells were fixed with acetone/methanol (1∶1) and stained using NDV-HIS and FITC conjugated anti-rabbit IgG (Sifin, Germany). Photos were taken with a Nikon ECLIPSE Ti-S camera and evaluated with NIS-Elements BR imaging software, measuring the area of 20 syncytia per virus, with two repetitions. For comparison of group differences, pairwise Wicoxon tests with Bonferroni adjustment were calculated. The family-wise type I error was set to 0.05. All calculations were performed using R, version 2.15.2 (2012-10-26), with package ‘exactRankTest’.

### Fluorescence and electron microscopy

For confocal microscopy, QM9 cells were seeded into 24-well plates on glass coverslips and infected at an moi of 5 with the respective viruses. After 1 h incubation on ice and 12 h incubation at 37°C and 3% CO_2_, cells were fixed with 3.7% formaldehyde in PBS, permeabilized with 0.1% Triton in PBS and blocked with 5% BSA in PBS. Subsequently, cells were incubated with a polyclonal monospecific antiserum against NDV-F protein [30], followed by washes with 5% BSA in PBS. Species specific secondary antibody labeled with FITC (Sifin) was added, followed by additional washing steps with 5% BSA in PBS and mounting in Mowiol. Images were collected on a Leica SP5 confocal microscope (Leica Microsystems GmbH, Wetzlar, Germany) using a 63× oil immersion objective with a numerical aperture of 1.4. Fluorochrome was excited using a 488-nm laser for FITC 488. For higher sensitivity, hybrid detectors were applied (HyD2, 488 nm). Sequential *z*-sections of stained cells were acquired for maximum projection. Images were processed using ImageJ software and Adobe Photoshop CS5 (Adobe systems).

For transmission electron microscopy, gradient purified and concentrated virions were adsorbed to formvar coated nickel grids for 7 min and then stained with phosphotungstic acid (PTA, pH 6.0).

### Determination of the intracerebral pathogenicity index (ICPI)

The ICPI of recombinant NDV was determined following European guidelines [31]. These animal experiments were approved by the animal welfare committee (Landesamt für Landwirtschaft, Lebensmittelsicherheit und Fischerei Mecklenburg-Vorpommern, Thierfelderstraße 18, 18059 Rostock, LALLF M-V/TSD/7221.3-1.1-053/10) and approved and supervised by the commissioner for animal welfare at the FLI representing the Institutional Animal Care and Use Committee (IACUC). Animals displaying severe clinical distress were sacrificed by exsanguination after knocking animals unconscious. Criteria for euthanasia were somnolence, apathy, akinesia or dyspnea.

## Results

Sequence determination of the R75/98 F gene (GenBank accession number KJ736742) yielded 1,792 nucleotides, which is in accordance with other NDV F genes. R75/98 F is encoded by an open reading frame (orf) of 1,662 bases and exhibits features like other NDV F proteins. Nucleotide sequence homology with the NDV Clone 30 F gene is 84.6%, and deduced amino acid homology 88.6%. The proteolytic cleavage site of R75/98 (aa 112–117) is characterized by five basic amino acids at the carboxy terminus of F2 and a phenylalanine at the amino terminus of F1 (^112^R-R-K-K-R*F^117^). Thus, it conforms to a pattern reminiscent of virulent NDV. The cleavage site is the major difference between the F proteins of lentogenic NDV Clone 30 (^112^G-R-Q-G-R*L^117^) and NDV R75/98. However, other differences are the substitution of a leucine (Clone 30) by an isoleucine (R75/98) within the leucine zipper motif (aa 281) between heptad repeat 1 and 2, as well as the last two amino acids of the cytoplasmic tail (CT) specifying ^552^K-M^553^ in Clone 30 and, ^552^R-T^553^ in R75/98. This variation leads to a secondary structure prediction that resulted in an additional turn at the very end of the R75/98 F protein.

### Generation of recombinant NDVs expressing chimeric fusion proteins

Recombinant NDV with chimeric F genes composed of NDV Clone 30 and NDV R75/98 sequences were used to investigate the influence of different F protein regions on pathogenicity. Six NDV/PPMV recombinants were constructed by exchanging either the complete gene or distinct parts of the gene encoding the F protein of lentogenic NDV Clone 30 by that of the mesogenic PPMV-1 R75/98 (Figure 1A). The full-length plasmid pflNDVFCSR75 contains the R75/98 cleavage site ^112^R-R-K-K-R*F^117^ in a Clone 30 backbone (Figure 1C), whereas pflNDVFR75 carries the whole R75/98 F gene (Figure 1B). Furthermore, four full-length plasmids carrying distinct parts of the R75/98 F gene in a Clone 30 backbone were constructed. Plasmid pflNDVFR75_1–117 contains the F_2_-encoding part of R75/98 F and the F_1_-encoding part of Clone 30 F. pflNDVFR75_1–527 encompasses nearly the whole F orf of R75/98 except sequences encoding the cytoplasmic tail. pflNDVFR75_112–553 carries the F_1_-encoding part of R75/98, and pflNDVFCSR75_528–553 encodes the R75/98 cytoplasmic tail. All recombinant F proteins possess the R75/98 cleavage site ^112^R-R-K-K-R*F^117^ (Figure 1D, E, F, and G). Recombinant viruses were rescued from all transfections indicating functionality of the chimeric F proteins. Sequencing of respective genome regions confirmed the identity of each virus.

### Expression and virion incorporation of the chimeric fusion proteins

**Figure 2:**
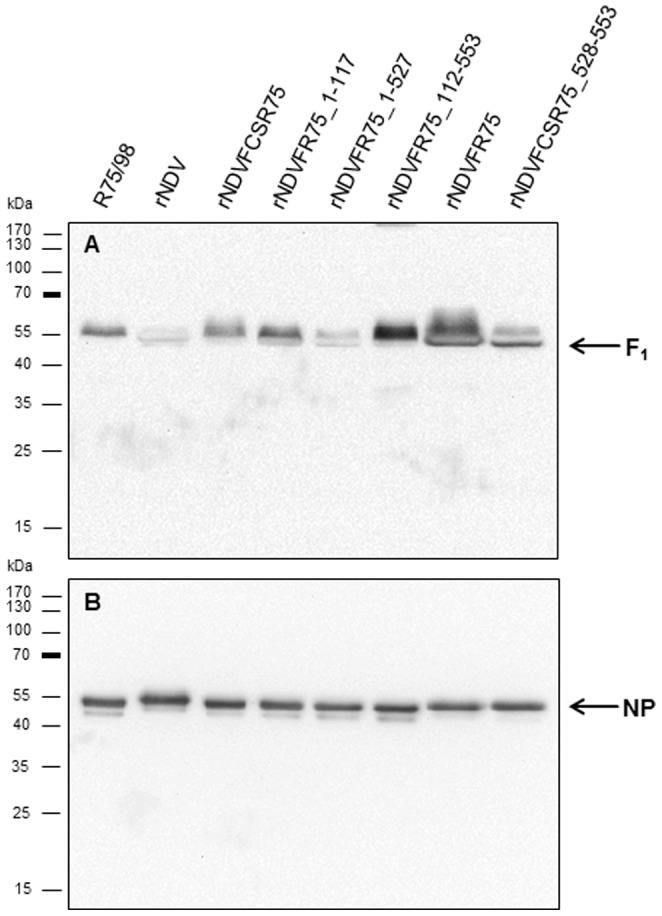
Western blot analysis of NDV recombinants. Purified virions with normalized protein levels (10 µg/lane) were separated by SDS-PAGE and blotted onto nitrocellulose. Membranes were incubated with polyclonal monospecific rabbit anti-NDV F serum (A) and monoclonal NDV-NP antibodies (B). Proteins were detected by chemiluminescence substrate (Pierce) after incubation with peroxidase-conjugated species-specific secondary antibodies.

Expression of chimeric F proteins and their incorporation into virions were determined in comparison to R75/98 and rNDV by Western blot analysis of purified virions using a polyclonal monospecific antiserum against the NDV-F protein (Figure 2A). The F protein was detected for wild type NDVR75/98, as well as for recombinant rNDV, rNDVFCSR75, rNDVFR75_1–117, rNDVFR75_1–527, rNDVFR75_112–553, rNDVFR75, and rNDVFCSR75_528–553 at a molecular mass of approximately 55 kDa, corresponding to the F1 subunit. Detection of the 12 kDa F2 subunit failed due to its small size or lack of reactivity of the antiserum. Detection of nucleoprotein (NP) as an authentic, unmodified NDV protein was carried out using monoclonal antibodies [13]. As expected, the NDV-NP protein was detected in all purified virions with a molecular mass of about 55 kDa (Figure 2B). Detection of the chimeric F proteins for all described purified recombinants confirms their expression and incorporation into the viral envelope.

### Replication of chimeric and parental viruses

**Figure 3:**
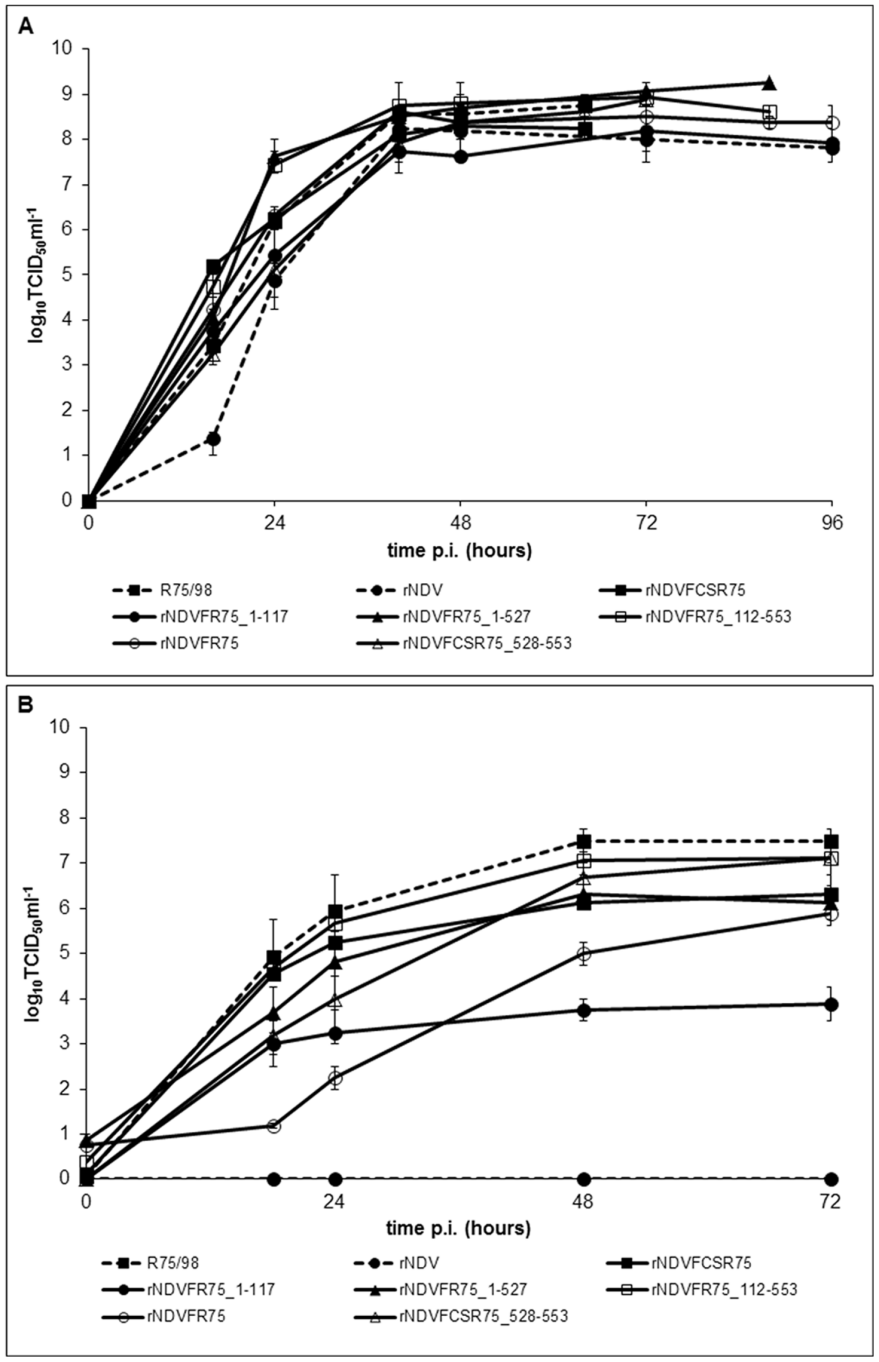
Kinetics of replication in ECE (A) and QM9 cells (B). SPF eggs were infected with 200 µl of 10^3^ TCID50 ml^−1^ and QM9 cells were infected at an moi of 0.01 with the respective viruses. Allantoic fluids of the eggs and cell culture supernatants were harvested at indicated time points and titers were determined by titration on QM9 cells, followed by immunofluorescence.

*In vivo* replication was examined in 10-day-old SPF ECEs, which were inoculated with 200 µl 10^3^ TCID_50_ ml^−1^ of the respective virus. All recombinant NDV with chimeric F protein replicated well. A slightly increased replication is evident within the first 24 h in comparison to parental rNDV. However, virus titers were comparable after 64 h and final titers reached about 10^8.0^–10^9.0^ TCID_50_ ml^−1^, demonstrating that the substitution of different regions of the Clone 30 F protein by those of R75/98 F has no significant effect on virus replication in ECE (Figure 3A). The *in-vitro* replication of parental and chimeric viruses was studied in QM9 and CEF. In comparison to ECE kinetics, replication of individual viruses in QM9 cells differed. While rNDVFR75_1–527, rNDVFCSR75, rNDVFR75_112–553, and R75/98 exhibited titers of about 10^5.0^–10^6.0^ TCID_50_ ml^−1^ already at 24 h post infection, rNDVFR75, rNDVFR75_1–117, and rNDVFCSR75_528–553 only replicated to titers of approximately 10^2.0^–10^4.0^ TCID_50_ ml^−1^ at this time point. However, final titers of most viruses reached about 10^6.0^–10^7.0^ TCID_50_ ml^−1^, whereas only rNDVFR75_1–117 produced a lower final titer of about 10^4^ TCID_50_ ml^−1^. As expected, parental rNDV did not replicate in QM9 cells because of its monobasic amino acid sequence at the F cleavage site and absence of trypsin-like proteases (Figure 3B). In CEF cells, most viruses also reached a similar final titer of 10^7.0±1.0^ TCID_50_ ml^−1^ at 72 h. However, recombinant rNDVFR75_1–117 again only produced a lower final titer of 10^5.0^ TCID_50_ml^−1^ (data not shown).

### Syncytium-assay

**Figure 4:**
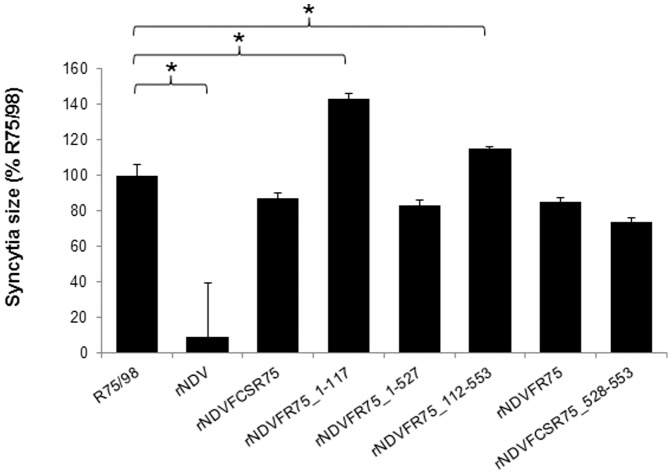
Syncytia size. BSR-T7 cells were infected at an moi of 0.01 with the respective viruses. At 24 h p.i. cells were fixed and syncytia of the recombinant viruses were visualized by immunostaining using a hyperimmune serum against NDV. The syncytia sizes were determined by measuring the areas of 20 syncytia per virus in triplicate assays with NIS-Elements BR imaging software. All values are expressed relative to R75/98 which had been set as 100%. Error bars represent standard deviations. Significant differences (P<0.05, Bonferroni correction) between R75/98 and recombinant viruses are indicated (*).

A typical property of virulent NDV is the fusion of plasma membranes of adjacent cells resulting in formation of polycaryocytes (syncytia) in cell culture. To examine if the different F proteins affect this process differently, a syncytium formation assay was performed in BSR-T7 cells (Figure 4). For parental virus rNDV, only infecting single cells due to its monobasic amino acid motif at the F protein cleavage site, the lowest value of infected cell size (9%) was measured compared to R75/98 which was set to 100%. All viruses possessing the R75/98 amino acid motif ^112^R-R-K-K-R*F^117^ formed syncytia, however to a different extent. Compared to R75/98, rNDVFCSR75_528–553 showed the lowest syncytia size (74%), while rNDVFR75_1–117 (143%) and rNDVFR75_112–553 (115%) exhibited significantly higher values. Three recombinants exhibited similar syncytia sizes, rNDVFCSR75 (87%), rNDVFR75 (85%) and rNDVFR75_1–527 (83%), which, however, were reduced in comparison to R75/98. These results indicate that the substituted regions of the F protein have an effect on syncytia formation.

### Fluorescence staining and electron microscopy

**Figure 5:**
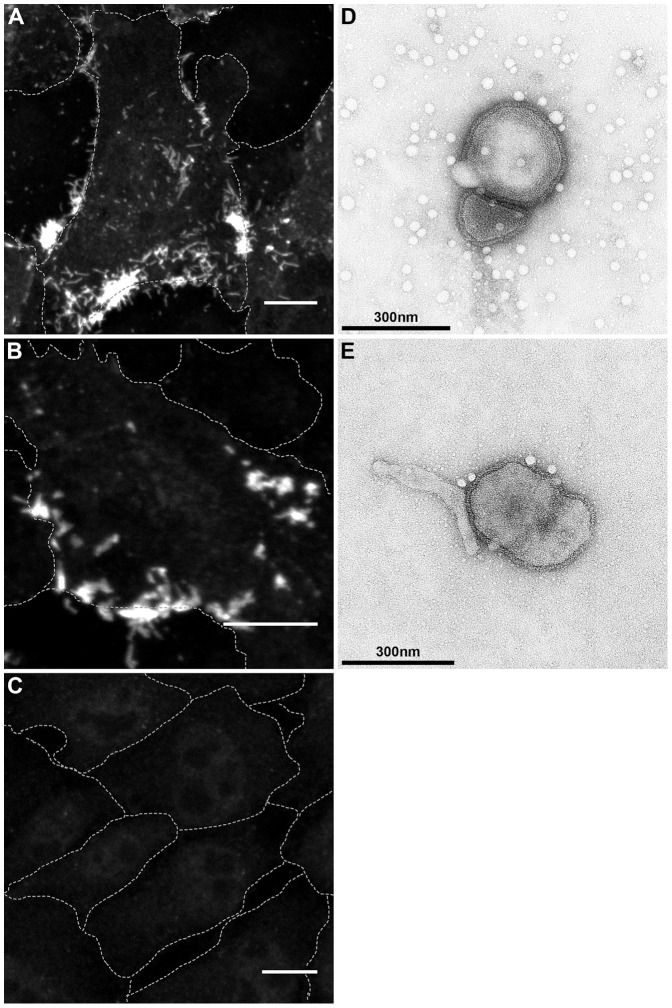
Fluorescence staining and electron microscopy. Expression and surface localization of the F protein of rNDV (A), rNDVFR75_1–117 (B) and mock infection (C) was analyzed by confocal microscopy after infection of QM9 cells with the respective viruses at an moi of 5. At 12 h p.i. cells were fixed and stained with a polyclonal monospecific antiserum against NDV-F protein (A–C, bars 10 µm, dashed lines represent cell borders). Virion morphology of rNDV (D) and rNDVFR75_1–117 (E) was analyzed by transmission electron microscopy after negative staining of purified virions with phosphotungstic acid.

Recombinant rNDVFR75_1–117 is characterized by a replication defect in CEF and QM9 cells but produced the largest syncytia (Figure 3, 4). However, no difference was detected in cells by indirect immunofluorescence and confocal microscopy using a polyclonal monospecific antiserum against NDV-F protein [30]. F proteins of R75/98, rNDV as well as of viruses expressing chimeric F were detected at the plasma membrane as exemplarily shown for rNDV and rNDVFR75_1–117 (Figure 5A–C). Furthermore, virus particles of rNDV and rNDVFR75_1–117 did not differ in shape or size from each other as demonstrated by transmission electron microscopy after negative staining (Figure 5D, E).

### Determination of virulence

ICPI in one-day-old chickens was determined to analyze the influence of the substituted F protein regions on virus pathogenicity. Parental rNDV and R75/98 exhibited ICPI values of 0.01 and 1.10, respectively, characterizing rNDV as lentogenic and R75/98 as mesogenic NDV. Recombinant rNDVFR75, expressing the F protein of R75/98, resulted in an unexpectedly low ICPI value of 0.6 (Figure 1B), despite presence of a polybasic amino acid sequence at the F cleavage site. Recombinant viruses with chimeric F protein showed ICPI values that characterize lentogenic or mesogenic NDV. In detail, rNDVFR75_112–553 can be classified as lentogenic with an ICPI of 0.59, whereas rNDVFCSR75, rNDVFR75_1–117, rNDVFR75_1–527 and rNDVFCSR75_528–553 revealed ICPI values of 1.36, 1.13, 1.36, and 1.13 respectively, which were even higher than that of the parental strain R75/98 but still of mesogenic pathotype (Figure 1C–G).

## Discussion

The F protein of NDV is one of the two surface glycoproteins. It mediates virus entry into the host cell by fusion of the viral envelope with the plasma membrane. The F protein is synthesized as a precursor protein of 553 amino acids which has to be activated by cleavage into F_1_ and F_2_ subunits which remain covalently linked by disulfide bonds [32]. This proteolytic cleavage is effected by host cell proteases [4] differing in their recognition amino acid sequence between monobasic and polybasic motifs. Thus, the amino acid sequence at the proteolytic cleavage site determines substrate specificity and, consequently, influences viral virulence [16], [33], [34]. Alteration of a monobasic amino acid sequence at the F protein cleavage site to a polybasic motif increased virulence of a lentogenic NDV to a mesogenic phenotype [22], [35], yet not to a fully virulent (velogenic) virus. Moreover, PPMV-1 strains which are virulent for pigeons may specify a polybasic cleavage site but lack pathogenicity for chickens [36]. Thus, the polybasic F protein cleavage site is not the sole determinant for NDV virulence.

We show here that insertion of the multibasic cleavage site from the mesogenic isolate R75/98 ^112^R-R-K-K-R*F^117^ into the lentogenic recombinant Clone 30 (ICPI 0.01) background resulted in a mesogenic virus (rNDVFCSR75) with an ICPI of 1.36. In contrast, replacement of the entire Clone 30 F gene by that of R75/98 produced a lentogenic NDV (rNDVFR75, ICPI 0.6), although, due to the presence of the polybasic proteolytic cleavage site, this virus is able to form syncytia in cell culture typical for NDV with polybasic cleavage sites. Modulation of virulence by exchange of heterologous F genes has also been reported recently [16]. F proteins of rNDVFCSR75 and NDV R75/98 exhibit an overall amino acid homology of 89.2% (493 of 553 amino acids are identical). To analyze the contribution of different regions of the F protein to virulence, specific regions (F_2_, ectodomain of F_1_, cytoplasmic tail) were exchanged between the two parental strains using Phusion PCR [29] for generation of full-length antigenome containing plasmids, followed by reverse genetics to generate virus recombinants that all specify the polybasic aa motif ^112^R-R-K-K-R*F^117^ of the PPMV-1 isolate R75/98. Phusion PCR is advantageous since it allows generation of tailored genome sequences without introduction of artificial restriction sites.

Our data demonstrate that, in addition to the proteolytic cleavage site, the F cytoplasmic tail (CT) is an important virulence determinant. Exchange of only the R75/98 CT by that of Clone 30 increased ICPI from 0.60 (rNDVFR75) to 1.36 (rNDVFR75_1–527) which was as high as that obtained by insertion of the polybasic cleavage site of R75/98 into the Clone 30 F protein (rNDVFCSR75). For paramyxoviruses it is known that the CT of surface glycoproteins interact with the matrix protein (M) and this interaction plays an important role in virus assembly, budding and release of infectious virus [37]–[40]. Moreover, NDV F may interact with NP and HN for incorporation into virus (like) particles [41]. Therefore, it is conceivable that interaction of F with internal M and/or NP is most efficient when these proteins originate from the same virus strain. Mutations and deletions within CT of NDV F resulted in altered phenotypes [42], also due to its role in virus assembly by directing the protein to cholesterol-rich membrane domains [43]. Since the CTs of R75/98 and Clone 30 differ in only two amino acids at the very end of the F protein, those two amino acids have to account for the observed differences in pathogenicity. Secondary structure predictions indeed indicated differences with R75/98 F CT forming an additional turn at the very end of the molecule.

It has been shown that specific regions of F and HN interact [44]. Recombinants rNDVFCSR75 and rNDVFR75_1–527 exhibited an identical ICPI of 1.36. This was not surprising since both viruses specify a polybasic amino acid sequence at the cleavage site in the F protein and contain homologous F ectodomains from the same origin. Thus, the homotypic interaction between F1 and F2 subunits appears to predominate over the interaction between F and HN, which originate from different parental viruses. Moreover, the interaction between the F ectodomain and HN is not altered by substitution of respective Clone 30 F regions by those of R75/98, at least in the presence of a polybasic cleavage site.

Whereas expression of chimeric F proteins within the NDV Clone 30 backbone had an effect on virus virulence, in Western blot analyses of purified virions no obvious differences were observed. The F_1_ subunit was detected in all viruses, confirming correct expression and virion incorporation of chimeric F. The uncleaved F_0_ precursor was not observed since virus originated from allantoic fluid, which contains proteases able to activate F protein irrespective of a poly- or monobasic amino acid sequence at its cleavage site. In contrast, differences became evident during viral replication in different cell types. Recombinant NDV without polybasic cleavage site neither replicated in QM9 cells (Figure 3B) nor formed syncytia in BSR-T7 cells (Figure 4), confirming that this virus is generally unable to replicate in cell cultures or to spread systemically in animal tissues without activation by trypsin-like proteases [45]. However, all recombinants expressing F proteins with the polybasic cleavage site ^112^R-R-K-K-R*F^117^ were replication-competent in cells and developed syncytia in BSR-T7 cells. It is notable that rNDVFR75_1–117 showed a replication defect in QM9 as well as in CEF cells, but formed the largest syncytia (143%) compared to parental R75/98. Development of such large syncytia results in an early cell death which may account for the decrease in viral titers resulting from an impairment of virus production over an infection period of 72 h. In contrast, final virus titers in ECE were comparable to the other virus recombinants with a polybasic F cleavage site supporting this assumption.

The F_2_ subunits of Clone 30 as present in rNDVFCSR75 and NDV R75/98 as in rNDVFR75_1–117 exhibit only 79.3% amino acid identity. Substitution of the Clone 30 F_2_ by that of R75/98 reduced pathogenicity from an ICPI of 1.36 in rNDVFCSR75 to 1.13 in rNDVFR75_1–117, indicating that this part of the protein also influences virulence. Unexpectedly, syncytia forming rNDVFR75_112–553 was characterized by an ICPI of 0.59 as obtained for rNDVFR75. This could be explained by a possible effect of the F_1_ subunit of R75/98.

Of relevance is the discrepancy between syncytia formation and low ICPI. The ability to form syncytia is controlled by the proteolytic cleavage site, whereas expression of a pathogenic phenotype requires the proteolytic cleavage site but additional virulence determinants. Thus, the detection of a polybasic motif at the cleavage site is not sufficient to identify APMV-1 as moderately or highly pathogenic and, consequently, can not form the sole basis for NDV diagnosis.

In conclusion, we demonstrated the importance of several regions of the F protein in modulating virulence of NDV. The most striking observation was the drastic increase in virulence by the exchange of the F cytoplasmic tail in a Clone 30 backbone by that of R75/98, with only two amino acid differences between mesogenic R75/98 and lentogenic Clone 30. This finding shows that only these two amino acids are responsible for the strikingly different phenotypes. This effect is most likely due to differences in the interaction of the F protein with internal viral proteins such as NP and/or M affecting virus assembly, budding and/or release but with no effect on virion morphology.

## References

[1] Alexander DJ (2000) Newcastle disease and other avian paramyxoviruses. Rev Sci Tech. 19:443–462. 10935273 10.20506/rst.19.2.1231

[2] International Committee on Taxonomy of Viruses (2012) In: Virus Taxonomy, Paramyxoviruses. King, A M Q, et al**.** (ed) Elsevier Academic Press San Diego, London. Pp 672–685.

[3] Czegledi A, Ujvari D, Somogyi E, Wehmann E, Werner O, et al (2006) Third genome size category of avian paramyxovirus serotype 1 (Newcastle disease virus) and evolutionary implications. Virus Res. 120(1–2):36–48. 16766077 10.1016/j.virusres.2005.11.009

[4] Lamb RA, Parks GD (2007) Paramyxoviridae: The viruses and Their Replication. In: Knipe DM,Howley PM, editors. Fields Virology. Philadelphia: Lippincott Williams& Wilkins, a Wolters Kluwer Business. pp.1449–1496.

[5] Steward M, Vipond IB, Millar NS, Emmerson PT (1993) RNA editing in Newcastle disease virus. J Gen Virol. 74 (Pt12):2539–2547. 8277263 10.1099/0022-1317-74-12-2539

[6] Alexander DJ (1998) Newcastle disease and other avian paramyxoviruses. A laboratory manual for the isolation and identification of avian pathogens. Kennett Square: American Association of Avian Pathologists. pp. 156–163.

[7] Alexander DJ, Wilson GW, Russell PH, Lister SA, Parsons G (1985) Newcastle disease outbreaks in fowl in Great Britain during 1984. VetRec. 117(17):429–434. 10.1136/vr.117.17.429 4071933

[8] Kaleta EF, Alexander DJ, Russell PH (1985) The first isolation of the avian PMV-1 virus responsible for the current panzootic in pigeons?. Avian Pathol. 14(4):553–557. 18766949 10.1080/03079458508436258

[9] Biancifiori F, Fioroni A (1983) An occurrence of Newcastle disease in pigeons: virological and serological studies on the isolates. Comp Immunol MicrobiolInfect Dis. 6(3):247–252. 10.1016/0147-9571(83)90017-6 6627911

[10] Dortmans JC, Rottier PJ, Koch G, Peeters BP (2011) Passaging of a Newcastle disease virus pigeon variant in chickens results in selection of viruses with mutations in the polymerase complex enhancing virus replication and virulence. J Gen Virol. 92(PT2):336–345. 20965986 10.1099/vir.0.026344-0

[11] Collins MS, Strong I, Alexander DJ (1996) Pathogenicity and phylogenetic evaluation of the variant Newcastle disease viruses termed “pigeon PMV-1 viruses” based on the nucleotide sequence of the fusion protein gene. Arch Virol. 141(3–4):635–647. 8645100 10.1007/BF01718322

[12] Alexander DJ (1997) Newcastle disease and other avian Paramyxoviridae infections. In: Calnek BW, editor. Diseases of poultry. Ames: Mosby-Wolfe Iowa State University Press. pp. 541–569.

[13] Werner O, Römer-Oberdörfer A, Köllner B, Manvell RJ, Alexander DJ (1999) Characterization of avian paramyxovirus type 1 strains isolated in Germany during 1992 to 1996. Avian Pathology. 28:79–88. 16147552 10.1080/03079459995082

[14] Deng R, Mirza AM, Mahon PJ, Iorio RM (1997) Functional chimeric HN glycoproteins derived from Newcastle disease virus and human parainfluenza virus-3. Arch Virol Suppl. 13 115–130. 10.1007/978-3-7091-6534-8_12 9413532

[15] Morrison TG (2001) The three faces of paramyxovirus attachment proteins. Trends Microbiol. 9(3):103–105. 11239770 10.1016/s0966-842x(01)01959-x

[16] Paldurai A, Kim SH, Nayak B, Xiao S, Shive H, et al (2014) Evaluation of the contributions of the individual viral genes to Newcastle disease virus virulence and pathogenesis. J Virol. 2014 Aug 88(15):8579–96. 24850737 10.1128/JVI.00666-14 PMC4135945

[17] Nagai Y, Klenk HD, Rott R (1976) Proteolytic cleavage of the viral glycoproteins and its significance for the virulence of Newcastle disease virus. Virology. 72(2):494–508. 948870 10.1016/0042-6822(76)90178-1

[18] Sakaguchi T, Matsuda Y, Kiyokage R, Kawahara N, Kiyotani K, et al (1991) Identification of endoprotease activity in the trans Golgi membranes of rat liver cells that specifically processes in vitro the fusion glycoprotein precursor of virulent Newcastle disease virus. Virology. 184(2):504–512. 1887586 10.1016/0042-6822(91)90420-g

[19] Glickman RL, Syddall RJ, Iorio RM, Sheehan JP, Bratt MA (1988) Quantitative basic residue requirements in the cleavage-activation site of the fusion glycoprotein as a determinant of virulence for Newcastle disease virus. J Virol. 62(1):354–356. 3275436 10.1128/jvi.62.1.354-356.1988 PMC250538

[20] Millar NS, Chambers P, Emmerson PT (1988) Nucleotide sequence of the fusion and haemagglutinin-neuraminidase glycoprotein genes of Newcastle disease virus, strain Ulster: molecular basis for variations in pathogenicity between strains. J Gen Virol. 69 (Pt3):613–620. 3351479 10.1099/0022-1317-69-3-613

[21] Collins MS, Bashiruddin JB, Alexander DJ (1993) Deduced amino acid sequences at the fusion protein cleavage site of Newcastle disease viruses showing variation in antigenicity and pathogenicity. Arch Viro.l 128(3–4):363–370. 10.1007/BF01309446 8435046

[22] Meulemans G (2002) Evolution of pigeon Newcastle disease virus strains. Avian Pathology. 31:515–519. 12427346 10.1080/0307945021000005897

[23] Peeters BP, de Leeuw OS, Koch G, Gielkens AL (1999) Rescue of Newcastle disease virus from cloned cDNA: evidence that cleavability of the fusion protein is a major determinant for virulence. J Virol. 73(6):5001–5009. 10233962 10.1128/jvi.73.6.5001-5009.1999 PMC112544

[24] Römer-Oberdörfer A, Mundt E, Mebatsion T, Buchholz UJ, Mettenleiter TC (1999) Generation of recombinant lentogenic Newcastle disease virus from cDNA. J Gen Virol. 80 (Pt11):2987–2995. 10580061 10.1099/0022-1317-80-11-2987

[25] Antin PB, Ordahl CP (1991) Isolation and characterization of an avian myogenic cell line. Dev Biol 143:111–121. 1985013 10.1016/0012-1606(91)90058-b

[26] Moscovici C, Moscovici MG, Jimenez H, Lai MMC, Hayman MJ, et al (1977) Continuous tissue culture cell lines derived from chemically induced tumors of Japanese quail. Cell. 11(1):95–103. 194709 10.1016/0092-8674(77)90320-8

[27] Buchholz UJ, Finke S, Conzelmann KK (1999) Generation of bovine respiratory syncytial virus (BRSV) from cDNA: BRSV NS2 is not essential for virus replication in tissue culture, and the human RSV leader region acts as a functional BRSV genome promoter. J Virol. 73(1):251–259. 9847328 10.1128/jvi.73.1.251-259.1999 PMC103829

[28] Schnell MJ, Mebatsion T, Conzelmann KK (1994) Infectious rabies viruses from cloned cDNA. EMBO J. 13(18):4195–4203. 7925265 10.1002/j.1460-2075.1994.tb06739.x PMC395346

[29] Geiser M, Cebe R, Drewello D, Schmitz R (2001) Integration of PCR fragments at any specific site within cloning vectors without the use of restriction enzymes and DNA ligase. Biotechniques. 31(1)::88–90, 92. 10.2144/01311st05 11464525

[30] Ramp K, Veits J, Deckers D, Rudolf M, Grund C, et al (2011) Coexpression of avian influenza virus H5 and N1 by recombinant Newcastle disease virus and the impact on immune response in chickens. Avian Dis. 55(3):413–421. 22017039 10.1637/9652-011111-Reg.1

[31] CEC (1992) Council Directive 92/66/EEC introducing Community measures for the control of Newcastle disease. Official Journal of the European Community. L 260:1–20.

[32] Morrison TG (2003) Structure and function of a paramyxovirus fusion protein. Biochim Biophys Acta. 1614(1):73–84. 12873767 10.1016/s0005-2736(03)00164-0

[33] Kawahara N, Yang XZ, Sakaguchi T, Kiyotani K, Nagai Y, et al (1992) Distribution and substrate specificity of intracellular proteolytic processing enzyme(s) for paramyxovirus fusion glycoproteins. J Gen Virol. 73 (Pt 3):583–590. 10.1099/0022-1317-73-3-583 1312118

[34] Nagai Y, Klenk HD (1977) Activation of precursors to both glycoproteins of Newcastle disease virus by proteolytic cleavage. Virology. 77(1):125–134. 841855 10.1016/0042-6822(77)90412-3

[35] Römer-Oberdörfer A, Veits J, Werner O, Mettenleiter TC (2006) Enhancement of pathogenicity of Newcastle disease virus by alteration of specific amino acid residues in the surface glycoproteins F and HN. Avian Dis. 50(2):259–263. 16863077 10.1637/7471-111505R.1

[36] Dortmans JC, Koch G, Rottier PJ, Peeters BP (2009) Virulence of pigeon paramyxovirus type 1 does not always correlate with the cleavability of its fusion protein. J Gen Virol. 90(PT11):2746–2750. 19641043 10.1099/vir.0.014118-0

[37] Takimoto T, Murti KG, Bousse T, Scroggs RA, Portner A (2001) Role of matrix and fusion proteins in budding of Sendai virus. J Virol 75(23):11384–11391. 11689619 10.1128/JVI.75.23.11384-11391.2001 PMC114724

[38] Takimoto T, Portner A (2004) Molecular mechanism of paramyxovirus budding. Virus Res. 106(2):133–145. 15567493 10.1016/j.virusres.2004.08.010

[39] Schmitt AP, Leser GP, Waning DL, Lamb RA (2002) Requirements for budding of paramyxovirus simian virus 5 virus-like particles. J Virol. 76(8):3952–3964. 11907235 10.1128/JVI.76.8.3952-3964.2002 PMC136107

[40] Waning DL, Schmitt AP, Leser GP, Lamb RA (2002) Roles for the cytoplasmic tails of the fusion and hemagglutinin-neuraminidase proteins in budding of the paramyxovirus simian virus 5. J Virol. 76(18):9284–9297. 12186912 10.1128/JVI.76.18.9284-9297.2002 PMC136449

[41] Pantua HD, McGinnes LW, Peeples ME, Morrison TG (2006) Requirements for the assembly and release of newcastle disease virus-like particles. J Virol. 80(22):11062–11073. 16971425 10.1128/JVI.00726-06 PMC1642154

[42] Samal S, Khattar SK, Paldurai A, Palaniyandi S, Zhu X, et al (2013) Mutations in the Cytoplasmic Domain of the Newcastle Disease Virus Fusion Protein Confer Hyperfusogenic Phenotypes Modulating Viral Replication and Pathogenicity. J Virol. 87(18):10083–10093. 23843643 10.1128/JVI.01446-13 PMC3754023

[43] Dolganiuc V, McGinnes L, Luna EJ, Morrison TG (2003) Role of the Cytoplasmic Domain of the Newcastle Disease Virus Fusion Protein in Association with Lipid Rafts. J Virol. 77(24):12968–12979. 14645553 10.1128/JVI.77.24.12968-12979.2003 PMC296069

[44] Gravel KA, Morrison TG (2003) Interacting domains of the HN and F proteins of newcastle disease virus. J Virol. 77:11040–11049. 14512552 10.1128/JVI.77.20.11040-11049.2003 PMC224984

[45] Panda A, Huang Z, Elankumaran S, Rockemann DD, Samal SK (2004) Role of fusion protein cleavage site in the virulence of Newcastle disease virus. Microb Pathog. 36(1):1–10. 14643634 10.1016/j.micpath.2003.07.003 PMC7125746

